# Class II Skeletal Malocclusion and Prevalence of Temporomandibular Disorders. An Epidemiological Pilot Study on Growing Subjects

**DOI:** 10.3390/jfmk6030063

**Published:** 2021-07-20

**Authors:** Grazia Fichera, Vincenzo Ronsivalle, Simona Santonocito, Khaled S. Aboulazm, Gaetano Isola, Rosalia Leonardi, Giuseppe Palazzo

**Affiliations:** 1 Department of General Surgery and Surgical-Medical Specialties, School of Dentistry, University of Catania, Via S. Sofia 78, 95124 Catania, Italy; graziafichera@hotmail.it (G.F.); vincenzo.ronsivalle@hotmail.it (V.R.); simonasantonocito.93@gmail.com (S.S.); gaetano.isola@unict.it (G.I.); gpalazzo@unict.it (G.P.); 2 Department of Biomedical and Dental Sciences and Morphofunctional Imaging, School of Dentistry, University of Messina, Via Consolare Valeria 1, 98123 Messina, Italy; 3 Department of Orthodontics, School of Dentistry Pharos University, Canal El Mahmoudia Street, Alexandria 21500, Egypt; k.aboulazm@pua.edu.eg

**Keywords:** Class II, temporomandibular disorder, crossbite

## Abstract

The purpose of our work is to evaluate the correlation between skeletal Class II malocclusion and temporomandibular disorders, by assessing potential different frequency scores compared with Class I and Class III skeletal malocclusion, and to evaluate associated etiological and risk factors. Fifty-five subjects were examined, 35 females and 20 males, with a mean age of 18 ± 1.3 years, divided into two groups: those with TMD and those without TMD, and prevalence was evaluated in the two groups of Class II subjects. Symptoms and more frequent signs were also examined in the TMD group. Regarding Group A (subjects with the presence of TMD), we found that 48% have a Class II, 16% have Class I, and 28% have Class III. In the totality of the group A sample, only 8% were male subjects. In Group B (subjects without TMD), we found that 40% were females, with 26.7% in Class I, 10% in Class II, and 3.3% in Class III; the male subjects in this group (60%) were distributed with 33.3% in Class I, 16.7% in Class II, and 10% in Class III. Class II malocclusion is not a causal factor of TMD but may be considered a predisposing factor.

## 1. Introduction

The second skeletal class represents a widespread disdain in the population and consequently a very frequent problem for orthodontists. This disdain is usually diagnosed based on occlusion, the relationship between the first molars, facial esthetics, and joint and chewing function. The malocclusion may be due to poor positioning on the sagittal plane of the jaw or both and may be affected by a vertical jaw dysplasia, mandibular dysplasia, or combined [1]. Examination of TMJ (temporomandibular joint) is important in the diagnosis of malocclusions; some characteristics of malocclusions can induce joint dysfunctions. It is estimated that in patients in the second skeletal class, the position of the condyle in the time pit, in habitual occlusion, can be normal, posterior, or anterior. The evaluation of this position is very important, and an altered position of the condyle can cause joint dysfunction [2]. The factors that can affect the balance of the stomatognathic apparatus and lead to a dysfunctional pathology of the temporomandibular joint can therefore be classified into predisposing, triggering, and perpetuating factors.

The most accredited etiological classification for temporo-mandibular dysfunctions (TMD) is that of the AAOP (American Academy of Orofacial Pain) Guidelines, developed by Okeson in 1996 [3].

The predisposing factors are represented by all those conditions that statistically increase the risk of breakage of the balance of the stomatognathic apparatus and therefore predispose to pathology: among these are some hereditary factors, such as the anatomy of the jaws, dental elements, articulation, and ligaments with a greater presence of elastic fibers (ligament laxity). The purpose of our work is to evaluate the correlation between a skeletal Class II and an articular dysfunction, researching the different frequency compared with the Class I and Class III etiology.

Given the very limited sample of subjects, our work is a pilot study, but it was intended to verify feasibility to be able to carry out a larger study later.

## 2. Materials and Methods

For the purpose of the present study, subjects were recruited from a retrospective cohort of patients treated at the Department of Orthodontics, University of Catania, Italy, between January 2017 and December 2020. The study was approved by the Institutional Human Ethics Committee, University of Catania (prof. AQAMDI, 09/28/20).

1. Inclusion Criteria
  (i) Patients with presence of skeletal Class I, Class II, or Class III;
  (ii) Patients who provided signed informed consent, according to the World.

Medical Association’s Declaration of Helsinki.

2. Exclusion Criteria
  (i) History of trauma;
  (ii) Previous orthodontic and/or gnathologic and/or physical therapies;
  (iii) Presence of further structural malformations in the areas of interest;
  (iv) Presence of uncontrolled systemic disease.

On the basis of these criteria, the sample comprised 55 individuals, 35 females and 20 males, with a mean age of 18 years.

The sample was divided into two subgroups: group A—patients with dysfunctions and group B—patients without dysfunctions. Group A comprised 25 patients, 23 females and 2 males, with a mean age of 18 ± 1.3 years.

**Figure 1:**
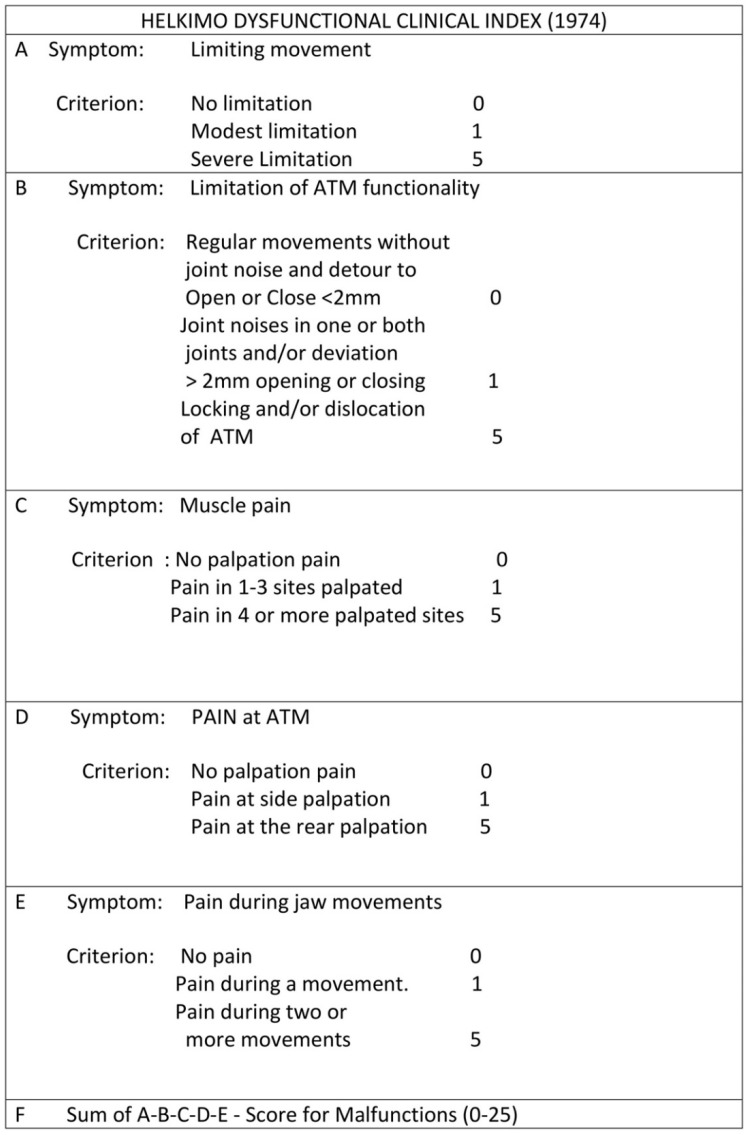
Helkimo clinical dysfunction index questionnaire.

Screening for craniomandibular dysfunctions was done by following the Helkimo dysfunction index guidelines [4] (Figure 1). Clinical examinations were perfumed by a single operator with 30 years’ experience in diagnosis and treatment of TMJ disorders (G.P.).

The same expert, blinded gnathologist performed all examinations. In particular, clinical examination of masticatory apparatus was performed using the Helkimo clinical dysfunction index (Di), which is based on five domains, each evaluating one of the following signs of TMJ (temporomandibular joint) dysfunction: limited TMJ mobility, limited TMJ function, jaw muscle pain to palpation, TMJ pain to palpation, and pain during mandibular movement. Jaw movements were evaluated to highlight the presence of any limitations. The temporomandibular joint was examined for the diagnosis of joint noise, taking into account possible deviations and deflections of the lower median line over three chewing cycles. Palpation of the chewing muscles and the temporomandibular joint was carried out, and the mandible excursions were examined to assess the presence of pain. Scores for each of the domains were based on the three-level scale of severity, i.e., 0 (no symptoms), 1 (mild symptoms), and 5 (acute symptoms), and were summed up to obtain a total dysfunction score, ranging from 0 to 25 points, with a high score indicating a higher temporomandibular dysfunction.

Group B comprised 30 individuals, 12 females and 18 males, with an average age of 18 ± 1.3 years. The cephalometric parameter considered for the evaluation of skeletal class was ANB angle; Class I (ANB 2 ± 2), Class II (ANB > 4), and Class III (ANB < 2). We also examined the percentage frequencies of the different TMJ pathologies found in Group A.

### Statistical Analysis

The data obtained from each examined subject were collected using a predefined data form. According to descriptive statistics, data were reported instead in the presence of specific variables, and the Chi-square test was used for inferential statistical evaluations. The odds ratio was also used to investigate the risk of TMD occurrence given the exposure to specific variables.

## 3. Results

Data obtained were primarily organized in tables and analyzed with descriptive statistics. Concerning Group A (subjects with the presence of TMD), we found that 48% (12 subjects) have a Class II, 16% (4 subjects) have Class I, and 28% (7 subjects) have Class III. Of the group A sample, only 8% were male subjects (Table 1). In Group B (subjects without TMD), we found that 40% were females 40% (12 subjects), with 26.7% (8 subjects) in Class I, 10% (3 subjects) in Class II, and 3.3% (1 subjects) in Class III; the male subjects in this group, 60% (18 subjects), were distributed with 33.3% (10 subjects) in Class I, 16.7% (5 subjects) in Class II, and 10% (3 subjects) in Class III (Table 2).

Group A was divided according to Helkimo’s index into three groups: DI (mild dysfunction), DII (high dysfunction), and DIII (severe dysfunction). We evaluated the frequency and percentage to find that 64% had mild dysfunction, 28% moderate, and only 8% of subjects had severe dysfunction (Table 3). According to the inferential statistics, the odds ratio was 5.31 in Group A and 0.91 in Group B. According to the chi-square test, statistically significant differences were found in the distribution of skeletal characteristics within Group A (*p* < 0.05), and no differences were detected in Group B (*p* > 0.05).

## 4. Discussion

In the last few decades, several studies have reported that the cause-and-effect and relationship between malocclusions and TMD are controversial. One of the first doctors to guess a relationship between occlusion and ATM was Costen (1934), who noted that many of his patients with pain in the ATM region, after changes in their occlusion, especially in the vertical dimension, significantly improved painful symptomatology [5]. Associations between some occlusions and TMD characteristics have been mentioned in many reports. Relationships were found between the open bite and TMD in some studies [6,7,8] and between the deep bite and TMD [9]. A significant association of TMD with monolateral inverse bite and midline displacement has also been reported [8]. Abnormal overbites and overjets may be associated with a wider deviation in the form of the time condyles, especially when combined with age, and this association has been interpreted as evidence to support the idea that long exposure to malocclusion may be associated with wider changes in ATM [10]. O’Ryan and Epker also presented that dentofacial deformities and malocclusions can lead to adaptive changes in ATM [11].

Schellas et al. hypothesized, based on the study of MRI images, that ATM pathology may be the cause of malocclusions and not vice versa. He concluded that it is extremely important before the treatment of a malocclusion, including orthognathic surgery, to evaluate any possible pathology of ATM [12]. Some studies have tried to relate the skeletal growth pattern to specific anatomical alterations [13]. Several studies have reported greater incidence of TMD in skeletal Class II (or excessive overjet) than in other toothless deformities, for example, in the third skeletal class [6,8,14,15,16,17]. A trend towards a higher incidence of TMD in patients with normal or low mandibular plane angles compared with patients with high mandibular plane angles has been observed [9,16]. There are also several studies reporting no significant associations between occlusal and TMD relationships. Some studies have failed to confirm significant relationships between ATM or muscle aching and skeletal class or between functional occlusal relationship and TMD [18]. In their review articles, Reynders et al. and Seligman and Pullinger concluded that there was no scientific evidence of a causal relationship between occlusion and TMD [19,20]. Wadhwa et al. studied three groups of patients, one with normal occlusion, one with untreated malocclusions, and one with orthodontically treated malocclusions. They concluded that the role of orthodontic treatment in improving or preventing TMD remains questionable [21].

Although Kirveskari and Alanen believe that there is sufficient evidence to justify the rejection of the hypothesis that occlusal factors are part of the causal complex of TM, it seems that, with the weak epidemiological data present, there is little predictive value in trying to prove that a single skeletal malocclusion is a specific risk for the development of a TMD [22]. In Class II therapy, it is necessary to implement an expansion of the maxillary [23,24,25], and from our data, many patients had a transverse deficit of the maxillary/crossbite, although no association between crossbite and TMD is proven [26]. From the data collected in the literature and supported by our observations, we found that many patients who had a skeletal Class II had a condylar dislocation posterior and meniscal anterior, and this can induce or aggravate a TMD. Although some patients with skeletal Class II had a normal or anterior condylar position [27]. This allows us to argue that the second skeletal class is a predisposing factor to the onset of TMD and that the physiological tolerance that allows the chewing system to overcome the action of pathogenic coxae unscathed is reduced when maxillo-mandibular orthopedic stability is reduced. The evidence available does not appear to be sufficient to justify the modalities of prophylactic therapy.

There are some limitations in the results of the present study that should be considered, such as the small sample of subjects. Moreover, this study encourages a larger study, even multicenter, to be able to say that there is a real correlation between Class II and TMD. Considering the recent enhancements in 3D imaging technology and digital superimposition methods of anatomical structures, more detailed studies are recommended [28,29,30].

In the light of the present findings, and from a clinical perspective, clinicians may also consider specific signs of malocclusion during the clinical examination of subjects affected by TMJ disorders.

## 5. Conclusions

According to the present findings, Class II malocclusion is not a causative factor of TMD but can be considered a predisposing factor.

## Figures and Tables

**Table 1:** Absolute frequency and percentage (n (%) values) of Group A in the different skeletal Classes.

Group A (N = 25)	Females 92% (N = 23)	Males 8% (N = 2)
Class I	4 (16%)	0
Class II	12 (48%)	1 (4%)
Class III	7 (28%)	1 (4%)

**Table 2:** Absolute frequency and percentage (*n* (%) values) of Group B in the different skeletal Classes.

Group B (N = 30)	Females 40%(N = 12)	Males 60% (N = 18)
Class I	8 (26.7%)	10 (33.3%)
Class II	3 (10%)	5 (16.7%)
Class III	1 (3.3%)	3 (10%)

**Table 3:** Absolute frequency and percentage (*n* (%) values) of Group A according to Helkimo index.

Group A TMD	Group A Prevalence (%) N = 25
DI	64% (16)
DII	28% (7)
DIII	8% (2)

## Data Availability

Data are available upon reasonable request.
